# Sociogenetic structure of *Polistes* (*Aphanilopterus*) *versicolor* Olivier, 1791 colonies (Hymenoptera, Vespidae, Polistini)

**DOI:** 10.1590/S1415-47572010005000081

**Published:** 2010-12-01

**Authors:** Keize Nagamati, Kimie Simokomaki, Caroline Vivian Gruber, Marco Antonio Del Lama

**Affiliations:** Departamento de Genética e Evolução, Universidade Federal de São Carlos, São Carlos, SPBrazil

**Keywords:** mating system, relatedness, multiple egg layers, territorial behavior, paper wasps

## Abstract

The observation of two distinct, well-defined oviposition areas in nests of the primitively eusocial wasp *Polistes versicolor* suggests the presence of multiple egg-layers and territorial behaviors. Electrophoretic analysis of enzyme loci in pupae from 35 colonies revealed an average observed heterozygosity of 0.10 and the existence of private polymorphisms, thereby indicating a low dispersion in this species. No evidence of diploid males was found. Phenotypic segregation analysis revealed the presence of more than one egg-laying female in 15 out of 35 colonies, as well as spatially preferential oviposition in 2 out of 13 nests, with distinct oviposition areas. Genetic relatedness estimates for brood were lower than expected for haplodiploid species under monogynous conditions (r = 0.75 for female broods and r = 0.5 for male) in 4 of those 13 nests, thereby inferring complex sociogenetic structuring in *Polistes versicolor* colonies*.*

## Introduction

The inclusion of the inclusive fitness concept in the Hamilton (1964) kin-selection model was a significant advance in the study of the evolution of eusociality. The Hamilton rule states that an altruistic trait can increase in frequency if rb > c, *i.e.*, the benefit (b) received by the donor's relatives, when weighted by their relationship (r) to the donor, exceeds the cost (c) of the action to donor fitness.

First proposed to explain eusocial behavior in insects, primarily hymenoptera, the kin-selection model was based on a monogamic reproductive system (monogyny-monandry), thus predicting high levels of intracolonial genetic relatedness. However, it is not uncommon to observe social species showing polyandry (egg-laying females mating with more than one male) or polygyny (more than one egg-laying female present in a colony), both resulting in lower levels of intracolonial genetic relatedness than predicted by the model. Although these observations and some alternative views (Wilson and Hölldobler, 2005) seem to go against the relevance of relatedness in the evolution of eusociality, its importance was recently restated by Hughes *et al.* (2008), who demonstrated monogamic behavior as being a basal characteristic in all eusocial lineages in Hymenoptera and, therefore, polygyny and polyandry would have emerged after these societies had been established.

Species of Vespidae are useful model organisms for studying the evolution of social behavior in insects, including wasps, wherein social organization ranges from solitary to eusocial (Hines *et al.*, 2007). When polygyny or serial polygyny (the substitution of the dominant egg laying females) occurs in social species of Vespidae, there is a subsequent modification in intranidal sociogenetic structure, thereby giving rise to complex conflicts between reproductive and non-reproductive females (West-Eberhard, 1969; Andersson, 1984; Pamilo, 1990).

In primitively eusocial societies of *Polistes*, colonies can be founded by a single (haplometrosis) or a group (pleometrosis) of females. When colonies are founded by an association of females, a hierarchical relationship is initially established through differential ovophagy by one of the females, and then definitively so through physical aggression against the remainder. Thus, one of the females becomes dominant (queen), whereas the remainder either turn into subordinates (workers) or leave the colony (Pardi, 1948; West, 1967; Itô, 1987).

Queens and workers of *Polistes* are morphologically similar (Eickwort, 1969; Evans and West-Eberhard, 1971). In a single colony of the tropical species *Polistes* (*Aphanilopterus*) *versicolor*, there is the likelihood of the ovaries being developed to a certain degree in about 40% of the females (K Simokomaki, unpublished data), denoting the coexistence of several potential egg layers in most colonies (Itô, 1993).

**Figure 1:**
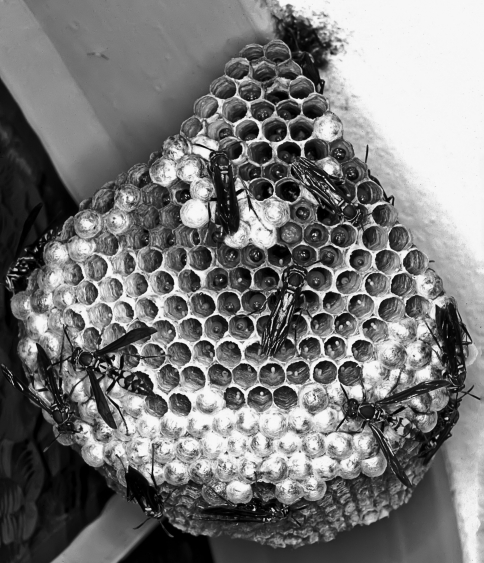
A photo of a *Polistes versicolor* nest with two distinct pupae brood areas.

Due to the natural occurrence of functional females in tropical species of *Polistes* (K Simokomaki, unpublished data), the observation of two oviposition sites separated by a space with empty cells, in the upper and lower parts of *P. versicolor* nests (Figure 1), gave rise to the hypothesis of polygyny and preferential oviposition by different females (territoriality). In the present study, the sociogenetic structure of *P. versicolor* colonies was established through the phenotypes of the brood for nine enzyme polymorphisms. There was ample evidence of polygyny and preferential oviposition in distinct areas of the nest, thereby revealing a complex sociogenetic structure.

## Methods

35 nests of *P.versicolor* Olivier, 1791 were collected in three towns in São Paulo State, Brazil, *i.e.*, 32 in São Carlos (22°02' S, 47°54' W, 14 from an urban area and 18 from the campus of São Carlos Federal University - UFSCar), two in Franca (20°32' S, 47°24' W), and one in Tambaú (21°29' S, 47°25' W), from April, 1993 to June, 1996, March to October 1998, and August, 2003. All were in the post emergence stage.

Each nest was first put into a plastic bag and removed from the support, to then be placed into a refrigerator for 10 min to reduce adult activity. The pupae were sequencially removed from the brood cells, placed in vials and then stored at -20 °C prior to analysis. The pupae from well-defined upper and lower areas of the nest were identified and accordingly grouped for this purpose.

Male and female pupae were individually homogenized in 0.2 mL of 0.2% 2-mercaptoethanol, and centrifuged at 5.000 g at room temperature for 10 min. The supernatants were subsequently used for electrophoresis analysis. 11 polymorphic loci from the 25 previously described enzyme loci (K Simokomaki, unpublished data) were selected for analysis, namely, esterases (*Est1, Est2*), phosphoglucomutases (*Pgm1, Pgm2*), isocitrate dehydrogenase (*Icd*)*,* β-hydroxybutyrate dehydrogenase (*Hbdh*), superoxide dismutase (*Sod1*), peptidase (*Pep-A*), leucylaminopeptidase (*Lap*), alcohol dehydrogenase (*Adh*) and α-glycerophosphate dehydrogenase (*Gpdh*). Horizontal electrophoresis was carried out in 14% corn starch gels (Penetrose 30, Corn Brazil) using either a Tris-citrate buffer (TC - 0.1 M Tris + 0.028 M citric acid, pH 7.5) or a Tris-citrate-borate buffer (TCB - 0.017 M Tris + 0.0023 M citric acid, pH 8.0, in the gel; 0.3 M boric acid, pH 8.3, in the electrodes). The electrophoretic run was carried out for either 5 h at a constant current of 3 mA/cm (TC), or 4 h at a constant current of 1.5 mA/cm (TCB). Detection of enzyme activity on the gel slabs was according to Harris and Hopkinson (1976) protocols.

Intra-locus heterozygosity was estimated based on the genotypes of female pupae. A chi-squared (χ^2^) homogeneity test (α = 0.05) was performed to test for differences in each genetic marker between observed and expected phenotype segregations in a monogyny-monandry system. Average intracolonial relatedness was calculated separately for both sexes by using Relatedness (v.5.08) (Queller and Goodnight, 1989), with colonies being equally weighted, and jackknifing over loci.

## Results

Genetic polymorphism was found at nine loci, inasmuch as only the electrophoretic variants α-Gpdh M and Icd S were detected in the colonies analyzed. Sod-2 electrophoretic variants were also detected. However, this variation was not considered because samples from only two colonies were analyzed. Electrophoretical analysis revealed two variants for Est-2, β-Hbdh, Sod-1, Adh and Pep-A (F and S) and three for Est-1, Pgm-1, Pgm-2 and Lap (F, M and S); these phenotypes were explained by two or three codominant alleles at the respective gene loci. The electrophoretic variants Lap S and Adh S were found only in pupae from nests collected in the UFSCar campus.

Heterozygosity of the polymorphic loci varied from 7.4% (locus *Lap*) to 49.8% (locus *Est-1*) (Table 1), with an average of 10.2%. None of the 337 male pupae collected from the 18 nests analyzed presented heterozygous phenotypes for any polymorphic loci.

As indicated through phenotypic segregation analysis, 15 of the 35 colonies were polygynous (Table 2). Phenotypic segregation of at least one locus in 10 colonies (nests 58, 63, 75, K30, 2.04, 2.12, 2.25, DQI, 2.02 and 2.03) was significantly different from the expected 1:1 ratio for monogyny-monandry, as exemplified by segregation of locus β-*Hbdh* in the broods from nests 58, 63 and 75. The presence of more than one egg-laying female in 10 of the 15 colonies (nests 58, 75, 2.01, DQI, K28, 114, 2.13, 2.11, 2.03 and 2.02) was inferred from noting three or more phenotypes for one enzyme loci, *e.g.* loci *Pgm-1* (colonies 75 and 2.11) and *Pgm-2* (colonies 114, 2.11 and 2.13).

**Figure 2:**
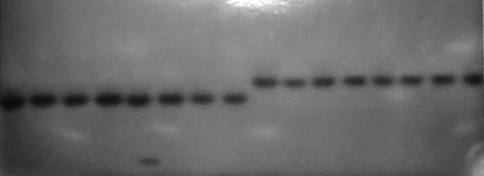
Starch gel showing β-hydroxybutyrate dehydrogenase (β-Hbdh) phenotypes in extracts of female (left) and male (right) pupae sampled in the lower and upper areas of nest 58 of *Polistes versicolor*.

There were two well-delimited oviposition areas in 13 of the 35 nests analyzed, with indications of polygyny in six. However, in only two did the segregation observed in broods collected from both areas indicate territoriality (Table 3). In nest 58, all the eight male pupae from the upper area presented a β-Hbdh F phenotype, whereas the β-Hbdh S phenotype was present in all the 18 female pupae from the lower area, thereby indicating brood-production by two distinct females (Figure 2). Likewise in nest 75, where β-Hbdh phenotypes from the pupae collected gave evidence of a preferential egg laying female in each area.

Genetic relatedness estimated for male broods in six of eight analyzed colonies (nests 58, 76, 144, 2.02, 2.04 and 2.05), was as expected (r = 0.5) for a monogynous colony, although it was higher than 0.7 in the other two (nests 75 and 82). No significant difference was found between genetic relatedness estimated for male broods from either the upper or lower areas.

On considering the estimated female genetic relatedness, it was noted that the values from four of the 12 colonies (nests 68, 82, 76 and 2.02) were distinct from that expected under monogamous conditions (r = 0.75). In five of the eight nests in which female broods from the upper and lower areas were analyzed, relatedness was found to be higher among brood from the lower area of the nest.

## Discussion

Although the analysis was specifically designed to determine intracolonial sociogenetic structure, certain population parameters were also estimated. The average heterozygosity was high, possibly a result of the non-random choice of enzyme loci for analysis. On considering the other 13 non-analyzed loci as monomorphic, with their inclusion in the analysis (K Simokomaki, unpublished data), an average heterozygosity of 0.102 would be obtained, which is high when compared to values reported for *Polistes* (0.026-0.084) by Lester and Selander (1979), and for primitively eusocial wasps (0.031 ± 0.006) by Graur (1985). The heterozygosity shown here is also high when compared to that reported by Metcalf *et al.* (1984) for *Polistes metricus* (H = 0.073) and *Polistes fuscatus* (0.065), and by Gaspar *et al.* (2007) for *Polistes satan* (H = 0.06). The observed heterozygosity in *P. versicolor* is particularly high when considering that it is a primitively eusocial species, where a lower genetic variation is expected than that in either solitary or highly eusocial species (Berkelhamer, 1983).

The private polymorphism at the *Lap* and *Adh* loci was found in brood from nests collected in the UFSCar campus, thereby inferring limited dispersion and consequently low gene flow in *P. versicolor*, as previously suggested by Del Lama and Ferreira (2003). The presumed low dispersion of male and female philopatry may explain the detection of private polymorphisms, despite the short distance between the sampled sites (an urban area of São Carlos and the UFSCar campus).

It is expected that low dispersion facilitates endogamous mating, thereby leading to an increase in homozygosis, even at the *csd* sex locus (Beye *et al.*, 2003). However, none of the males presented a heterozygous phenotype in any of the analyzed loci, thus indicating the absence of diploid males in the colonies of *P. versicolor*. This result differs from the reports of diploid males in other species of *Polistes* (Tsuchida *et al.*, 2002; Liebert *et al.*, 2004). Additional studies are necessary in order to identify the mechanisms of difficult endogamous mating in this species.

Apart from females of this species usually mating with only one male (monandry) (N Gobbi – personal communication; Strassmann, 2001), the observed phenotypic segregations in the analyzed loci revealed the presence of more than one egg-laying female in about 40% of the *P. versicolor* colonies. However, in one-third of these, the observed polygyny may be attributed to random oviposition by yet another female, or even others, in the colony. This supposition agrees with observations that dominant queens partially lose control of the colony in those *P.versicolor* nests with numerous cells, thus resulting in occasional oviposition (N Gobbi, Personal communication). The elevated numbers of cells (from 170 to 488 cells) in the analyzed nests gives additional support to this hypothesis.

Differential distribution of enzyme phenotypes was apparent in the broods collected from only 2 of the 13 *P. versicolor* nests with two distinct oviposition areas, thus supporting the hypothesis of oviposition by distinct females (Table 3).

Territorial behavior, with the establishment of territories controlled by a single queen, has been reported in *Polistes simillimus* nests with both a high number of cells and paragynic social organization (Pamilo, 1991; Gobbi *et al.*, 1993). A like situation occurred in *Polistes canadensis canadensis* (West-Eberhard, 1986). The author noted that, in mature colonies, the established queen remained in the lower regions of the nest containing new cells, whereby oviposition was frequent. Concomitantly, competition among eventual new egg-layers was confined to the upper areas with recycled cells.

Our results add support to observations made by West-Eberhard (1986), since the levels of genetic relatedness were found to be higher among broods from the lower areas of the nests analyzed. For instance, in nest 58, the relatedness of female broods located in the upper area was 0.14 ± 0.22, whereas this was 0.55 ± 0.23 in the lower. A similar situation occurred in nest 73 (Table 3). Thus, with the dominant queen laying eggs in the lower area (her own territory), the average expected relatedness in future female broods would thus be 0.75, as would also be the case of a single new egg-laying female in the upper area of the nest. However, the latter situation would change on more than one female starting to lay eggs in the same area, thereby leading to a reduction in brood genetic relatedness. This situation would continue until the establishment of a new dominant female.

Genetic relatedness in most of the analyzed colonies was higher than that reported for *P. satan* (0.384 ± 0.069 *vs.* 0.405 ± 0.088) (Gaspar *et al.*, 2007), as well as for *P. versicolor* (0.371 ± 0.084) and *P. canadensis* (0.339 ± 0.097) (Strassmann *et al.*, 1989). Lower genetic relatedness is expected in polygynous colonies, although values over 50% may occur in this instance, if one single female is responsible for most of the eggs. Another scenario would be if queens are highly related to each other (Hughes *et al.*, 1993), which is likely to occur in *Polistes* where the nest foundation is frequently the undertaking of an association of sisters.

The large number of empty cells and the occurrence of spatially discontinuous oviposition areas may be signs of the existence of more than one egg-laying female and territoriality in *P*. *versicolor* nests, where each female and her associates take care of their respective broods. Under these conditions, kin recognition among individuals that interact in the colony is facilitated by territorial behavior, which may compensate for the reported low kin-recognition ability among nestmates in *Polistes* colonies (Gamboa *et al.*, 1986; Panek and Gamboa, 2000).

The enzyme phenotypes of the brood did not lend support to expectations of detecting polygyny and territoriality in some nests. This, however, may be due to a deficiency of the marker used to identify genetic differences between individuals from the two distinct areas. Nevertheless the observed heterozygosity was inadequate for completely ascertaining genetic relationships among nestmates. Due to the relevance of territorial behavior in understanding intranidal sociogenetic structure, a re-analysis using highly polymorphic loci, such as microsatellites, is necessary to obtain more robust evidence of territoriality in post-emergence *P. versicolor* nests. This re-analysis should also include pre-emergence nests, thereby allowing for a complete picture of the sociogenetic structure of colonies within different developmental stages. Intranidal territoriality may be more common than reported here, and thus this reanalysis could possibly contribute to a better understanding of the genetic structure of these territories. Moreover, it would be an aid in determining conditions favorable to this behavior and its relevance to sociality in this species.

## Figures and Tables

**Table 1:** Allele frequencies (p), observed intra-locus heterozygosity (Hobs) and average heterozygosity (H) in electrophoretically analyzed pupae of *Polistes versicolor*. N represents the number of colonies sampled.

Loci	N	Allele	p	H_obs_
*Est-1*	31	*F*	0.305	
		*M*	0.297	
		*S*	0.398	0.498
*Est-2*	23	*F*	0.924	
		*S*	0.076	0.151
*Hbdh*	27	*F*	0.326	
		*S*	0.674	0.391
*Sod-1*	28	*F*	0.554	
		*S*	0.446	0.324
*Pep-A*	15	*F*	0.717	
		*S*	0.283	0.185
*Pgm-1*	25	*F*	0.112	
		*M*	0.804	
		*S*	0.084	0.370
*Pgm-2*	25	*F*	0.221	
		*M*	0.358	
		*S*	0.421	0.269
*Lap*	13	*F*	0.004	
		*M*	0.934	
		*S*	0.062	0.074
*Adh*	8	*F*	0.670	
		*S*	0.330	0.296
			H	0.102

**Table 2:** Allozymic phenotypes observed in pupae progenies indicating polygyny (due to the number of phenotypes or to the phenotypic segregation analysis) in 15 *Polistes versicolor* colonies.

Nest	Sex	Loci	Phenotype	N	χ^2^		Nest	Sex	Loci	Phenotype	N	χ^2^
58	Female	*Hbdh*	F	3			2.02	Female	*Sod-1*	F	3	
			FS	21	13.5					S	3	
	Female	*Sod-1*	F	1						FS	4	
			S	8				Female	*Est-1*	F	2	
			FS	15						M	4	
	Female	*Pgm-2*	F	15						FM	4	
			M	1				Male	*Lap*	M	77	
			FM	2						S	20	33.5

63	Female	*Est-1*	FS	1	10.3			Male	*Pgm-2*	F	29	
			S	13						S	72	18.3

	Female	*Hbdh*	FS	1	10.3		2.03	Female	*Sod-1*	F	5	
			S	13						S	2	
	Female	*Sod-1*	FS	1	10.3					FS	8	
			S	13					*Lap*	M	10	

75	Male	*Hbdh*	F	4						S	2	5.3

			S	23	13.4		2.11	Female	*Hdbh*	F	3	
	Female	*Pgm-1*	F	1						S	4	
			M	2						FS	3	
			FM	1				Female	*Pgm-1*	F	1	
	Male	*Pgm-1*	F	6						M	6	
			M	21	7.0					FM	3	

2.01	Female	*Sod-1*	F	3						MS	2	
			S	9						FS	1	
			FS	1				Female	*Pgm-2*	F	6	
	Female	*Lap*	M	20						S	3	
			S	2						FS	4	

			FM	1			2.12	Male	*Pgm-2*	F	1	
			MS	7						S	18	15.2

K30	Male	*Pgm-2*	F	1			2.13	Female	*Hbdh*	F	2	
			S	7	4.5					S	4	

2.04	Male	*Pgm-2*	F	12						FS	2	
			S	2	7.1			Female	*Pgm-2*	F	1	

DQI	Female	*Sod-1*	F	22						S	6	
			FS	4	12.46					FS	3	

	Male	*Sod-1*	F	9			2.25	Female	*Est-2*	F	2	
			S	1	6.4					S	15	9.9
	Female	*Pep-A*	F	6				Female	*Pgm-2*	S	15	
			FS	6						FS	2	9.9

			S	6			114	Female	*Pgm-2*	M	1	
	Female	*Hbdh*	FS	2						S	2	
			SS	16	10.89					MS	1	

	Female	*Adh*	FF	2			K28	Female	*Sod-1*	F	3	
			FS	12						S	1	
			SS	4						FS	4	

**Table 3:** Observed phenotypes in pupae from two different areas in nests of *Polistes versicolor*, evidencing the presence of a different egg layer in each oviposition site.

Nest	*Loci*	Area	Sex			
58	*Hbdh*			F	S	FS
		U	Female	3		3
		
		L	Male	**8**		
			Female		**18**	

				F	S	FS
		U	Female		2	
		
75	*Hdbh*		Male		**9**	
		L	Female		1	1
			Male	**4**	**14**	

**Table 4:** Genetic relatedness (r, 1st line) and standard error (SE, 2^nd^ line) in brood collected in the upper (U) and lower (L) areas in nests of *Polistes versicolor*. The number of pupae used to estimate r is indicated on the 3^rd^ line.

Nest		58	60	63	64	69	75	76	82	108	144	2.02	2.04	2.05
		0.46	-	-	-	-	0.91	0.64	0.78	-	0.60	0.42	0.29	0.59
	Male	0.24	-	-	-	-	0.10	0.24	0.23	-	0.24	0.16	0.25	0.26
U		8	-	-	-	-	9	6	7	-	12	52	4	5
	
		0.25	0.73	0.86	0.80	0.70	0.75	0.01	1.00	0.61	0.41	-	0.63	-
	Female	0.26	0.21	0.07	0.14	0.35	0.19	0.62	0	0.10	0.25	-	0.08	-
		6	16	7	5	8	2	2	4	8	3	-	25	-
	
		-	-	-	-	-	0.70	0.66	0.80	-	0.59	0.47	0.35	0.76
	Male	-	-	-	-	-	0.21	0.24	0.21	-	0.25	0.19	0.15	0.16
L		-	-	-	-	-	18	15	19	-	15	83	9	11
		0.64	0.73	0.97	0.73	0.66	0.67	-	-	0.69	-	0.25	0.63	-
	Female	0.16	0.25	0.03	0.26	0.45	0.19	-	-	0.08	-	0.11	0.08	-
		18	16	7	11	7	2	-	0	7	-	9	15	-

		0.46	-	-	-	-	0.76	0.66	0.80	-	0.58	0.45	0.31	0.62
	Male	0.24	-	-	-	-	0.18	0.24	0.21	-	0.26	0.18	0.14	0.22
U + L		8	-	-	-	-	27	21	26	-	27	135	13	16
	
		0.49	0.70	0.90	0.74	0.70	0.59	0.01	1.00	0.64	0.52	0.25	0.63	-
	Female	0.15	0.25	0.06	0.22	0.37	0.24	0.62	0	0.08	0.22	0.13	0.09	-
		24	32	14	16	15	4	2	4	15	3	9	40	-
